# Immune Reactions in Behçet's Disease

**DOI:** 10.1155/2014/985689

**Published:** 2014-06-02

**Authors:** Fumio Kaneko, Dongsik Bang, Rafi Haner Direskeneli, Shigeaki Ohno, Yoshiaki Ishigatsubo

**Affiliations:** ^1^Institute of Dermato-Immunology and Allergy, Southern TOHOKU Research Institute for Neuroscience, Koriyama, Japan; ^2^Department of Dermatology and Cutaneous Biology Research Institute, Yonsei University College of Medicine, Seoul, Republic of Korea; ^3^Devision of Rheumatology, School of Medicine, Marmara University Hospital, Marmara University, Istanbul, Turkey; ^4^Ophthalmology, Hokkaido University Graduate School of Medicine, Sapporo, Japan; ^5^Department of Internal Medicine and Clinical Immunology, Yokohama City University Graduate School of Medicine, Yokohama, Japan

Behçet's disease (BD) is a systemic and chronic inflammatory disorder which mainly involves mucocutaneous, ocular, vascular, gastrointestinal, and/or central nervous system organs in association with some genetic background as the intrinsic factors. Generally, the disease starts with oral recurrent aphthous stomatitis (RAS) in young-aged generation and develops to the systemic recurrent inflammation. BD patients are considered to be mainly distributed from the countries around the Mediterranean, old Silk-Road, and Korea to Japan. Historically, there was the antiquity description by Hippocrates and the disease was precisely reported by Turkish dermatologist, Hûlusi Behçet, as a trisymptom complex in 1937. BD pathogenesis is still obscure and classified as an autoimmune and recently in kind of autoinflammatory disorders. To clarify the genetics, epidemiology, diagnosis, pathogenesis, and treatment, a number of investigative studies must be addressed. In this special issue, we approached the clinicopathology of BD in aspects of the quality of life (QOL) in the oral health, a trial of new diagnostic ways utilized by the hypersensitivity to oral streptococci, infectious immunology in correlation with heat shock protein (HSP), T-helper 1(Th1), and Th17 cell responses in the inflammatory lesions and abnormal immune response to herpes simplex virus (HSV).

One of the papers of this issue addresses oral health QOL (OHQOL) in comparison with BD patients and non BD patients having RAS as controls, because RAS is sometimes seen in even healthy children and adults. However, OHQOL of BD patients is worse in their life activities because of more frequent and long involvements though it is not correlated with HLA-B51 gene. Another paper describes that BD patients have hypersensitivity to streptococcal group bacteria. To make a diagnosis for BD, observation of the clinical manifestations and mysterious “Pathergy test” by a thick stick-like 20G syringe needle are conventionally performed. However, the positive rate by the stick test is low in BD patients lately and the diagnostic value for BD is suspected. Then, the authors indicate the high diagnostic value by a fine stick with self-saliva, because oral streptococci are found to be included. The oral streptococci are considered to be one of the extrinsic triggering factors for BD patients with or without HLA-B51 gene. The authors speculate the relationship between the oral immune reaction and the systemic manifestations. In other papers, the following immunological phenomena are described. HSP60/65, which might be derived from mycobacterium and/or streptococci infection and are considered to work as a scavenger for the damaged tissues, might play an important role in innate immunological reactions in BD pathogenesis. They are also speculated to transfer some antigenic peptides to the antigen presenting cells (APCs) through toll-like receptors (TLRs) which activate specific T-cells and enhance MHC-peptide complexes. The mechanism of both Th1 and Th17 cells was influenced by interferon-*γ*, which produce interleukin 17 (IL-17) found in autoimmune disorders, is addressed in BD lesions. In another paper, the authors review the role of HSV in the immunopathogenesis of the HSV-induced mouse model because HSV DNA is detectable from the lesions of BD patients with the clinical evidence supporting the role of HSV infection.

The 5 papers propose the clinical characteristics and pathogenesis of BD which have been better clarified. The real pathogenesis of BD still remains to be obscure as far as the questions are unclear in the correlation between the intrinsic factors like genetic background and the extrinsic triggering factors. However, the number of BD patients seems to be decreasing and the clinically severe cases seen before have been released by the recent excellent immunological treatments.

*Fumio Kaneko* *Fumio Kaneko* *Dongsik Bang* *Dongsik Bang* *Rafi Haner Direskeneli* *Rafi Haner Direskeneli* *Shigeaki Ohno* *Shigeaki Ohno* *Yoshiaki Ishigatsubo* *Yoshiaki Ishigatsubo*

